# Gyro-Free Inertial Navigation Systems Based on Linear Opto-Mechanical Accelerometers

**DOI:** 10.3390/s23084093

**Published:** 2023-04-19

**Authors:** Jose Sanjuan, Alexander Sinyukov, Mohanad F. Warrayat, Felipe Guzman

**Affiliations:** 1 Department of Aerospace Engineering, Texas A&M University, College Station, TX 77843, USA; 2 Department of Physics and Astronomy, Texas A&M University, College Station, TX 77843, USA

**Keywords:** accelerometers, inertial navigation, gyro-free inertial navigation system

## Abstract

High-sensitivity uniaxial opto-mechanical accelerometers provide very accurate linear acceleration measurements. In addition, an array of at least six accelerometers allows the estimation of linear and angular accelerations and becomes a gyro-free inertial navigation system. In this paper, we analyze the performance of such systems considering opto-mechanical accelerometers with different sensitivities and bandwidths. In the six-accelerometer configuration adopted here, the angular acceleration is estimated using a linear combination of accelerometers’ read-outs. The linear acceleration is estimated similarly but requires a correcting term that includes angular velocities. Accelerometers’ colored noise from experimental data is used to derive, analytically and through simulations, the performance of the inertial sensor. Results for six accelerometers, separated by 0.5 m in a cube configuration show noise levels of 10${}^{-7}$ m s${}^{-2}$ and 10${}^{-5}$ m s${}^{-2}$ (in Allan deviation) for time scales of one second for the low-frequency (Hz) and high-frequency (kHz) opto-mechanical accelerometers, respectively. The Allan deviation for the angular velocity at one second is $10^{-5}$ rad s${}^{-1}$ and $5\times 10^{-4}$ rad s${}^{-1}$. Compared to other technologies such as MEMS-based inertial sensors and optical gyroscopes, the high-frequency opto-mechanical accelerometer exhibits better performance than tactical-grade MEMS for time scales shorter than 10 s. For angular velocity, it is only superior for time scales less than a few seconds. The linear acceleration of the low-frequency accelerometer outperforms the MEMS for time scales up to 300 s and for angular velocity only for a few seconds. Fiber optical gyroscopes are orders of magnitude better than the high- and low-frequency accelerometers in gyro-free configurations. However, when considering the theoretical thermal noise limit of the low-frequency opto-mechanical accelerometer, $5\times 10^{-11}$ m s${}^{-2}$, linear acceleration noise is orders of magnitude lower than MEMS navigation systems. Angular velocity precision is around $10^{-10}$ rad s${}^{-1}$ at one second and $5\times 10^{-7}$ rad s${}^{-1}$ at one hour, which is comparable to fiber optical gyroscopes. While experimental validation is yet not available, the results shown here indicate the potential of opto-mechanical accelerometers as gyro-free inertial navigation sensors, provided the fundamental noise limit of the accelerometer is reached, and technical limitations such as misalignments and initial conditions errors are well controlled.

## 1. Introduction

Inertial navigation systems (INS) are an essential part of applications where information about localization and orientation is necessary [1,2,3,4]. Their use has been rapidly growing in recent years, especially within the field of autonomous and unmanned vehicles where global navigation satellite systems (GNSS) or detailed maps are not available [5,6]. These scenarios are broad and diverse and include planetary exploration, space missions in deep space, underwater navigation, and tunnel exploration, among others. INSs can be combined with vision-based methods to improve accuracy, especially at long time scales, and provide initial conditions. Usually, INSs are based on triaxial accelerometers and gyroscopes, which measure the linear acceleration and the angular velocity of a rigid body, respectively. Alternatively, an array of linear accelerometers can provide linear and angular acceleration estimates as well. They are known as gyro-free inertial navigation systems (GF-INS) and have been of interest over the last 60 years [7,8,9,10,11,12,13,14,15,16,17,18]. Their benefits over traditional INSs are, in general, lower complexity, cost, weight, volume, power consumption, and higher reliability. Furthermore, using only one type of sensor eases integration, interfaces, and calibration. The performance of a GF-INS depends on the accelerometer performance, number, and arrangement. At minimum, six distributed uniaxial units are necessary to fully determine the motion and orientation of a rigid body. Among these minimal configurations, the ones avoiding the need to solve unstable nonlinear differential equations are of special interest [12,13,15,16,17].

In this paper, we investigate the performance of such configurations when using uniaxial opto-mechanical accelerometers [19,20]. We adopted the six-accelerometer scheme proposed in [12,15]. A higher number of accelerometers will yield better accuracyl however, we are interested in the performance of GF-INS in its simplest configuration. For the analysis and simulations, we consider the noise of opto-mechanical accelerometers that we have developed and characterized experimentally. Our analysis is carried out in the frequency domain and relies on the propagation of the power spectral density of the accelerometer’s noise. A similar approach has been recently published for conventional strapdown inertial navigation [21]. However, to our knowledge, this analysis is lacking for gyro-free inertial navigation. This method allows us to identify and quantify the coupling between angular and linear acceleration measurements and predict the performance of the system for a given accelerometer’s noise spectrum. Systematic errors [22] due to bias instability, scale factors, or mounting misalignments are not considered, and will be addressed briefly elsewhere. However, given the nature of the opto-mechanical accelerometers, we foresee misalignments and misplacements of the accelerometers, the main source of concern. Similarly, we assume that the initial conditions of position, orientation, velocity, and angular velocity are perfectly known.

The paper is organized as follows. First, the opto-mechanical accelerometers are briefly described, and their experimentally determined noise spectrum is presented. The GF-INS configuration, the navigation equations, and the noise propagation through the system are described in Section 3. Time-domain simulations’ are discussed in Section 4 and compared to the analytical results presented in Section 3. We end by comparing the performance of GF-INS based on opto-mechanical resonators to existing inertial sensors and gyroscope technologies in Section 5, followed by a discussion, summary, and future prospects in Section 6.

## 2. Accelerometer Description and Noise Models

**Figure 1:**
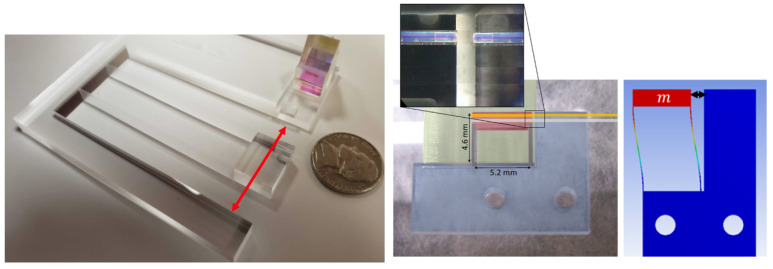
Left: low-frequency (LF) opto-mechanical resonator [23]. Red arrow indicates the sensitive axis. Right: High-frequency (HF) opto-mechanical resonator [19].

Opto-mechanical accelerometers [19,20,23] are fused silica mechanical resonators, where a test mass is held by two 100 μm thick parallel leaf strips. Accelerations induce motions on the test mass, which are measured using heterodyne interferometry [24,25] or fiber-based optical cavities [26,27]. Below the natural angular frequency of the mechanical resonator, $\omega_{0}$, acceleration, and displacement are linearly related as $x\left(t\right)=\omega_{0}^{-2}\ddot{x}\left(t\right)$. Thus, the resonance frequency defines the sensitivity and bandwidth of the accelerometer: small $\omega_{0}$ implies high sensitivity but low bandwidth, and vice versa. We have chosen two types of opto-mechanical resonators for this analysis: a high-bandwidth one with $\omega_{0}/2\pi$∼10 kHz and a high-sensitivity one with $\omega_{0}/2\pi$∼ 5 Hz. They are shown in Figure 1. In the following, we refer to them as high-frequency (HF) and low-frequency (LF) accelerometers.

**Figure 2:**
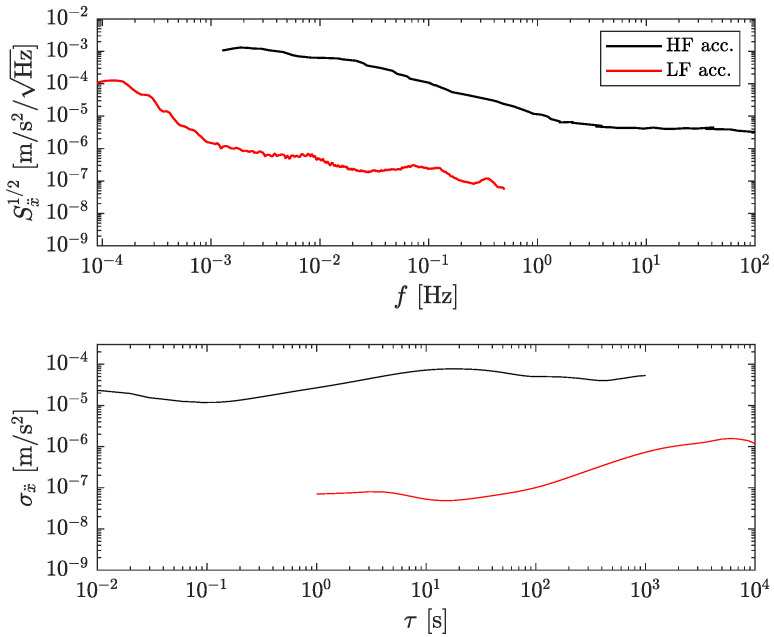
Measured accelerometers’ noise levels. Top: square root of the PSD. Bottom: overlapping Allan deviation. HF: high-frequency accelerometer (black). LF: low-frequency accelerometer (red). The curve for the HF accelerometer represents its actual noise. The curve for the LF accelerometer includes signal and noise and thus indicates a conservative upper limit of its actual noise curve.

The experimental noise curves for both accelerometers are shown in Figure 2 as the square root of the power spectral density (PSD) and as the overlapping Allan deviation. Note the rather high bandwidth and high noise levels of the HF accelerometer (in black) compared to the LF accelerometer (in red). The noise curve of the HF accelerometer represents its actual performance, which is limited by the optical read-out [19]. However, the noise curve of the LF accelerometer includes noise and signal since it is complicated to isolate the accelerometer from ground vibrations at such low frequencies. The curve, thus, represents a very conservative upper limit of the actual limiting noise. For instance, the expected thermal noise limit of the LF accelerometer is about $5\times 10^{-11}/\sqrt{f}$ m s${}^{-2}/\sqrt{\mathrm{Hz}}$, i.e., 1000 smaller at 1 Hz than the noise used here [23].

## 3. Gyro-Free Inertial Navigation System Configuration

**Figure 3:**
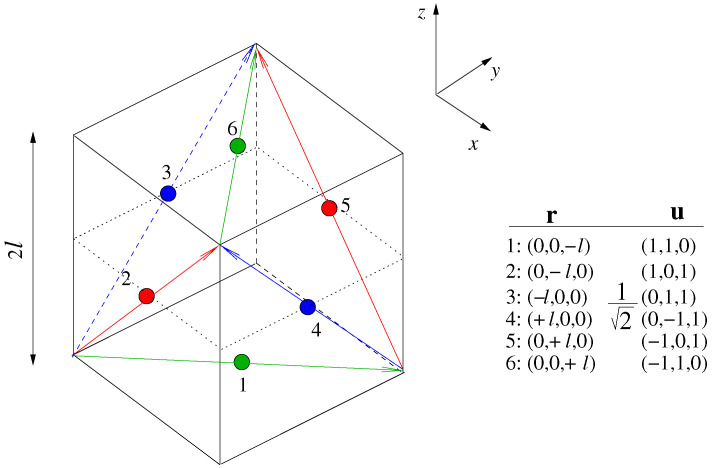
GF-INS cube configuration [12,15,16]. Six uniaxial accelerometers are placed on the faces of a cube of size 2*ℓ* with their sensitive axes oriented across the diagonals of each face. r and u are the accelerometers’ positions and directions, respectively. They are assumed to be free of errors.

The GF-INS is based on six uniaxial linear accelerometers placed and oriented in a cube of size 2*ℓ* as shown in Figure 3. The accelerometers are located in the center of the faces of the cube with sensitive directions along the diagonals. This particular configuration allows the estimation of angular acceleration with a linear combination of accelerometers’ readings. Angular velocity, Ω, is then readily obtained by numerical integration. The linear acceleration, $\ddot{r}$, is estimated as a linear combination of accelerometers’ readings plus correcting terms, including angular velocities. Details can be found in [12,16].

The accelerations at the sensors’ locations are

(1) $${\ddot{r}}_{i}={\ddot{r}}_{\mathrm{CoM}}+\dot{\Omega }r_{i}+\Omega^{2}r_{i}$$

where Ω is the skew-symmetric matrix

(2) $$\begin{array}{c} \Omega =\left(\begin{array}{ccc} 0 & -\Omega_{z} & +\Omega_{y}\\ +\Omega_{z} & 0 & -\Omega_{x}\\ -\Omega_{y} & +\Omega_{x} & 0 \end{array}\right) \end{array}$$

where $\Omega_{x,y,z}$ are the angular velocities around the axes *x*, *y*, and *z*, respectively. $r_{i}$ values are the locations of the accelerometers with respect to the center of mass (CoM) of the rigid body. The accelerometers’ readings, $A_{i}$, are the projections of the actual accelerations onto the accelerometers’ sensitive axes, $u_{i}$—see Figure 3:

(3) $$A_{i}={\ddot{r}}_{i}\cdot u_{i}.$$

The angular accelerations are calculated as

(4) $$\left(\begin{array}{c} {\hat{\dot{\Omega }}}_{x}\\ {\hat{\dot{\Omega }}}_{y}\\ {\hat{\dot{\Omega }}}_{z} \end{array}\right)=\frac{1}{2\sqrt{2}\ell }\left(\begin{array}{c} +A_{1}-A_{2}+A_{5}-A_{6}\\ -A_{1}+A_{3}-A_{4}-A_{6}\\ +A_{2}-A_{3}-A_{4}+A_{5} \end{array}\right),$$

where we use the notation ^ to indicate estimates. Subsequently, the linear accelerations are calculated as

(5) $$\left(\begin{array}{c} {\hat{\ddot{r}}}_{x}\\ {\hat{\ddot{r}}}_{y}\\ {\hat{\ddot{r}}}_{z} \end{array}\right)=\frac{1}{2\sqrt{2}}\left(\begin{array}{c} +A_{1}+A_{2}-A_{5}-A_{6}\\ +A_{1}+A_{3}-A_{4}+A_{6}\\ +A_{2}+A_{3}+A_{4}+A_{5} \end{array}\right)+\ell \left(\begin{array}{c} {\hat{\Omega }}_{y}{\hat{\Omega }}_{z}\\ {\hat{\Omega }}_{x}{\hat{\Omega }}_{z}\\ {\hat{\Omega }}_{x}{\hat{\Omega }}_{y} \end{array}\right)$$

where the angular velocity ${\hat{\Omega }}_{x,y,z}$ is calculated by integrating the angular acceleration from Equation (4), assuming the initial angular velocity is known. Finally, the position and orientation of the body are estimated by double integration of the linear and angular accelerations, respectively.

### 3.1. Accelerometers’ Noise Propagation

In the following, we estimate the GF-INS performance considering the noise of the accelerometers introduced in Section 2. While the estimation of the angular acceleration performance is straightforward, the linear acceleration is less obvious due to the presence of the angular velocity terms—see Equation (5). We assume the six linear accelerometers exhibit the same noise levels and that they are all uncorrelated. We denote PSDs as S(ω), and its square-rooted version as $S^{1/2}\left(\omega \right)$, where ω=2πf with *f* the Fourier frequency. The PSD of the uniaxial accelerometer noise symbol is $S_{n}$ and has units of ($m\;s^{-2}{)}^{2}$/Hz. From Equation (4), the angular acceleration noise is (in PSD):

(6) $$S_{{\hat{\dot{\Omega }}}_{x,y,z}}\left(\omega \right)=\frac{1}{8\ell^{2}}S_{n}\left(\omega \right),$$

which, as expected, is inversely proportional to $\ell^{2}$. The noise in the linear acceleration is, in general,

(7) $$S_{\hat{\ddot{x}}}\left(\omega \right)=\frac{1}{2\pi }\int R\left(\tau \right)\exp\left\{-i\omega \tau \right\}d\tau$$

where

(8) $$R\left(\tau \right)=E[{\hat{\ddot{r}}}_{x}\left(t\right){\hat{\ddot{r}}}_{x}\left(t+\tau \right)]$$

is the autocorrelation function of linear accelerations with *noisy* accelerometers. Equation (7) can be approximated to

(9) $$S_{\hat{\ddot{x}}}\left(\omega \right)≃\frac{1}{2}\left[S_{n}\left(\omega \right)+\frac{1}{2\ell^{2}}S_{\int Adt\int Adt}\left(\omega \right)\right],$$

where

(10) $$S_{\int Adt\int Adt}\left(\omega \right)=\int S_{\int A_{i}dt}\left(\omega \right)S_{\int A_{j}dt}\left(\omega -\omega^{\prime }\right)\frac{d\omega^{\prime }}{2\pi },$$

and

(11) $$S_{\int Adt}\left(\omega \right)=\frac{S_{A}\left(\omega \right)}{\omega^{2}}=\frac{S_{n}\left(\omega \right)}{\omega^{2}}.$$

**Figure 4:**
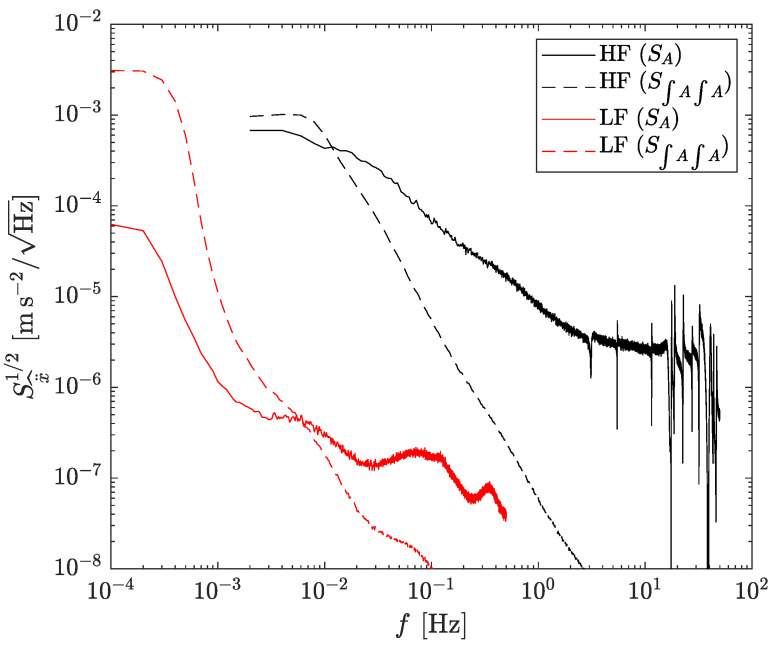
Predicted linear acceleration noise when using HF (black) and LF (red) accelerometers. Solid traces are the first term in Equation (9), while dashed traces are the contributions of the cross terms due to leakage from angular velocity noise. The effect becomes dominant for f< 6 mHz in the LF optomechanical resonator, and barely visible in the HF accelerometer.

The term $S_{\int Adt\int Adt}$ is the PSD of the product in the time domain of two random variables (noisy components in ${\hat{\Omega }}_{i}$ and ${\hat{\Omega }}_{j}$), which in the frequency domain corresponds to the convolution of their PSDs [28]. $S_{\int Adt}$ is the PSD of the noise of the accelerometer converted to linear velocity. The first term in Equation (9) indicates that the combination of six accelerometers reduces the noise compared to a single accelerometer due to the averaging of four accelerometers. The second term is the noise from the angular velocity, which couples in a non-linear fashion, and it is inversely proportional to the square of the cube size, $\ell^{2}$. In the following, *ℓ* is set to 0.25 m, which is considered a reasonable size for GF-INSs in vehicles or spacecraft. The linear acceleration noise predictions for both accelerometers, LF and HF, are shown in Figure 4. For the latter, the noise leakage of the angular velocity onto the linear acceleration has a minor effect. For the former, the effect dominates for frequencies below ∼6 mHz and grows steeply towards lower frequencies. Note that the noise levels described in this section, and shown in Figure 4, are estimated assuming only the inherent noise of the accelerometers, i.e., in absence of actual angular acceleration. The effects of the latter are described in Section 4.2.

## 4. Simulations

The noise predictions from the previous section have been validated by time-domain simulations. They have been implemented as follows. Linear and angular accelerations are applied to the rigid body. Next, the accelerations in the locations of the accelerometers are calculated and projected onto the accelerometers’ sensitive axes, as described in Section 3. Subsequently, the accelerometers’ noise models are added to the signals. The noise is generated by coloring Gaussian white noise using digital filters [29]. At this point, the accelerometers’ readings including signals and noise are available, and the equations and algorithms to recover the linear and angular acceleration are implemented. Equation (4) is used to estimate angular accelerations, ${\dot{\Omega }}_{x,y,z}$. The output is, in turn, numerically integrated to estimate $\Omega_{x,y,z}$. Linear accelerations are calculated using Equation (5) and are then integrated to obtain linear velocity and position. Similarly, angular velocity is integrated to obtain orientation. The simulations for the high-frequency accelerometer were run at sampling rate $f_{s}$ = 100 Hz and lasted for 500 s. The low-frequency accelerometer sampling rate was 1 Hz, and the simulations lasted for about 10,000 s. For all simulations, we assumed a cube size of *ℓ* = 0.25 m—see Figure 3.

**Figure 5:**
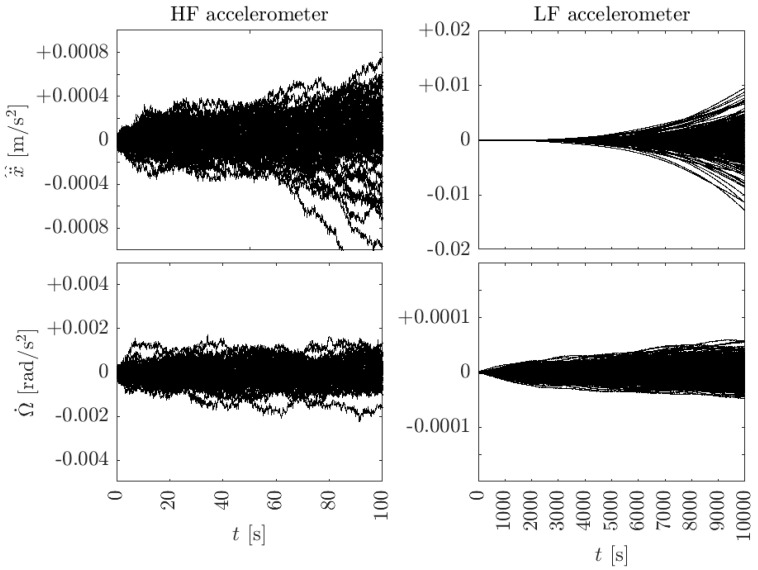
Time–domain simulation results for linear (**top**) and angular (**bottom**) accelerations. Left: high-frequency accelerometer. Right: low-frequency accelerometer, where the effect of angular velocity leakage into linear acceleration is clearly visible after 2000 s. A total of 1000 independent simulations are shown where no force or torque was applied to the body.

Results in the time domain are shown in Figure 5 for the linear and angular accelerations and for both accelerometers. For this simulation, no external forces or torques were applied. Thus, for noiseless accelerometers, the outputs should be zero. In both cases, 1000 independent realizations were carried out. The left side shows the results of the HF accelerometer. Linear and angular accelerations are shown in the top and bottom figures, respectively. Angular acceleration remains stationary, as expected from Equation (6). On the contrary, linear acceleration diverges slowly after ∼50 s. The reason, as described in Section 3.1, is due to the presence of angular velocity noise coupling into the linear acceleration calculation. The plots on the right show the low-frequency accelerometer case. Clearly, linear acceleration is strongly corrupted by the angular velocity noise for t>2000 s. The error in the angular acceleration remains bounded, similarly to the HF case.

**Figure 6:**
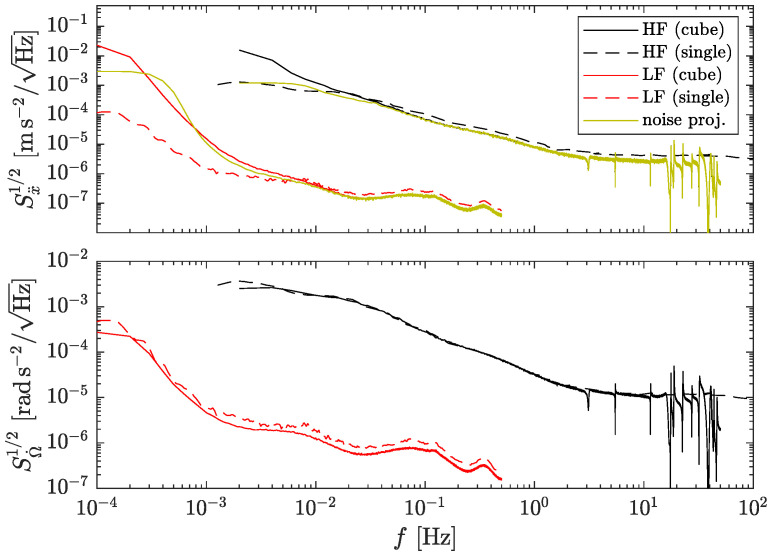
Top: Linear acceleration performance. Bottom: angular acceleration. Cube indicates GF-INS configuration with *ℓ* = 0.25 cm, while single corresponds to the acceleration noise of an optomechanical accelerometer, i.e., the same curves shown in Figure 2. HF: high-frequency. LF: low-frequency.

The results in the frequency domain are shown in Figure 6. The top plot shows the square root of the PSD for the linear acceleration, while the bottom plot corresponds to the angular acceleration. Solid traces are the results from the simulations. Dashed traces on the top figure indicate the linear acceleration noise of a single accelerometer, i.e., the same curves given in Figure 1. The ocher traces in the top plot are the theoretical noise predictions from Section 3.1. Clearly, the linear acceleration worsens at low frequencies compared to a single accelerometer due to the excess angular velocity noise—cf. Equation (9).

### 4.1. Position and Orientation Uncertainties as a Function of Time

**Figure 7:**
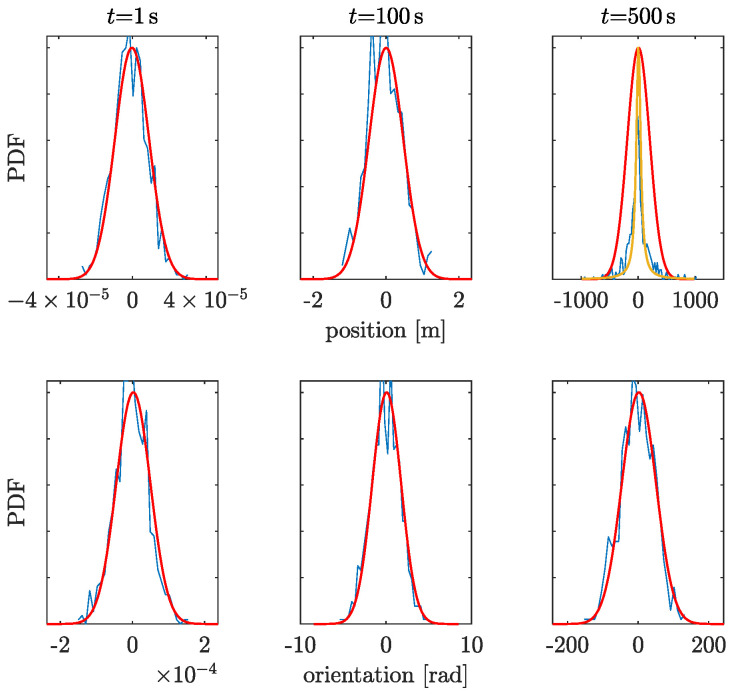
High−frequency accelerometer position (**top**) and orientation (**bottom**) uncertainties for different times. Results are shown as probability density functions (PDF) calculated from 1000 simulations. Blue: simulation results. Red: Gaussian distribution fit. Ocher: Cauchy distribution fit.

**Figure 8:**
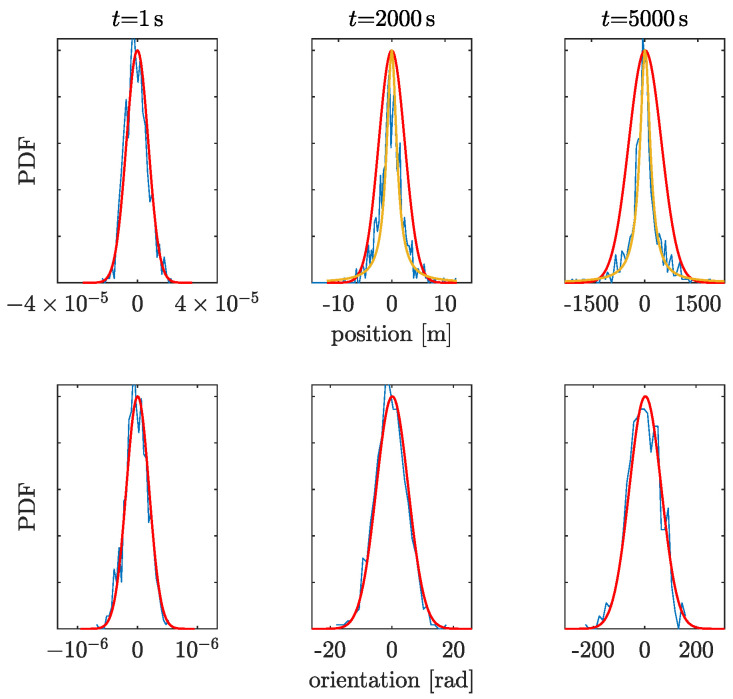
Same as Figure 7 for the low-frequency accelerometer.

In GF-INSs, the position and orientation are calculated by integrating twice the linear and angular accelerations, which cause the uncertainties to grow quickly with time. Figure 7 and Figure 8 show the probability density functions (PDF) of the position and orientation uncertainties at different times for both accelerometers in the GF-INS configuration. The PDFs of the orientation (bottom panels in Figure 7 and Figure 8) remain Gaussian, as expected from Equation (4), since the accelerometer’s noise was assumed to be Gaussian. However, the distribution of the linear acceleration remains Gaussian only until a certain time. After that, the angular velocity leaks into the linear acceleration and dominates the position uncertainty. At this point, the PDF gradually morphs into another distribution, which we have fitted to a Cauchy distribution. For the high-frequency accelerometer, the change in distribution occurs at t> 500 s, while for the low-frequency accelerometer, the morphing occurs at about 2000 s.

**Figure 9:**
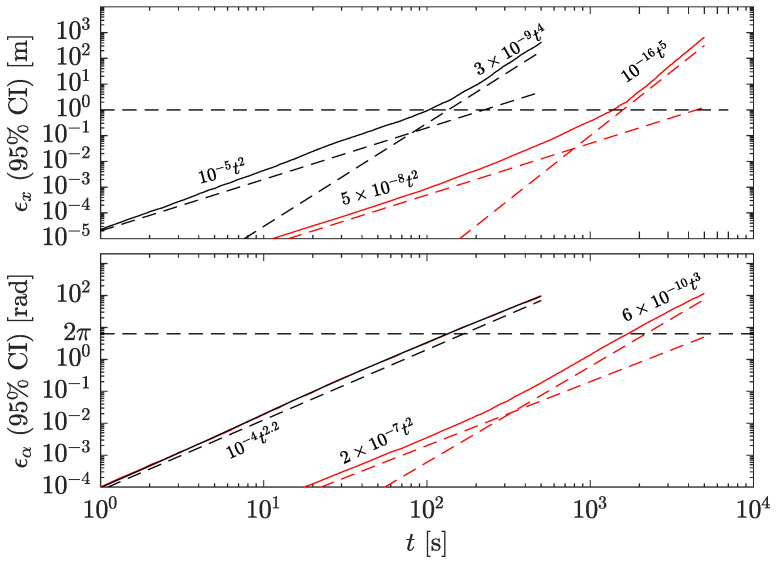
Uncertainty (95% probability) as a function of time in position (**top**) and orientation (**bottom**) for the HF (black) and LF (red) GF-INSs. Dashed lines indicate 1 m and 2π rad errors and are used as reference.

Figure 9 shows the position (top) and orientation (bottom) uncertainties within a 95% probability as a function of time calculated from the PDFs. The change in slope in the linear acceleration corresponds to the change in PDF distribution, while the change in the trend in the orientation is related to the colored noise of the accelerometer. The dashed line in the position’s plot indicates an error of one meter, which for the HF and LF accelerometers happens after about 100 and 1000 s, respectively. After that, the acceleration error grows as $t^{4}$ and $t^{5}$ for the LF and HF accelerometers, respectively. The dashed trace for the orientation’s plot indicates an uncertainty of 2π rad, which occurs at 200 and 2000 s for the HF and LF accelerometers, respectively.

### 4.2. Simulations with Signals

**Figure 10:**
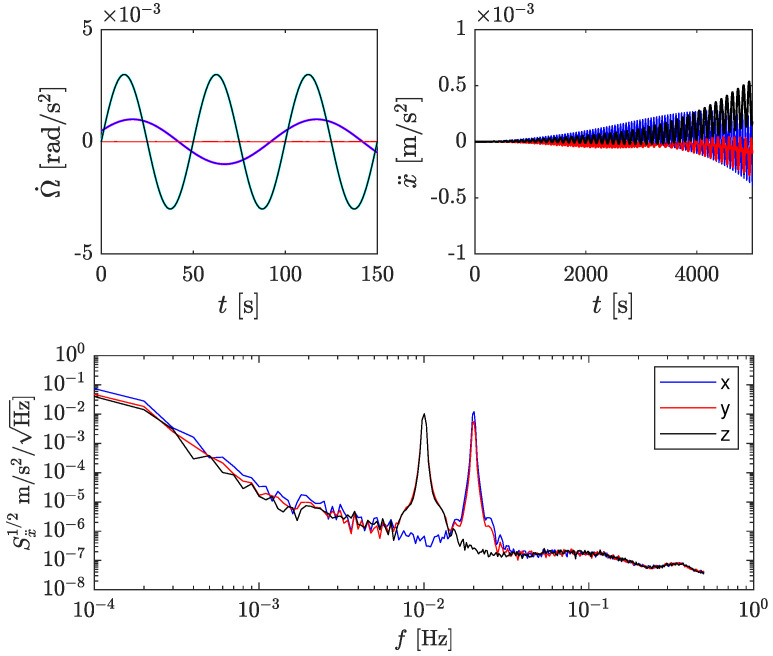
Simulation results when injecting angular accelerations. (**Top left**): recovered angular acceleration. (**Top right**): recovered linear acceleration, where the effect of the angular velocity is clearly visible. (**Bottom**): linear acceleration square root of the PSD. The peaks at 10 and 20 mHz appear due to the angular velocity cross-talk.

In this section, we present simulations’ results when applying angular accelerations to the body, as opposed to the previous section, where the body was at rest. The results are shown only for the LF accelerometer, but the conclusions apply to the HF accelerometer as well. The applied angular accelerations to the rigid body were

(12) $$\begin{array}{ccc} \;\;{\dot{\Omega }}_{x} & = & {\dot{\Omega }}_{0,x}\sin\left(2\pi \omega_{x}t+\phi_{x}\right) \end{array}$$

(13) $$\begin{array}{ccc} {\dot{\Omega }}_{y} & = & 0\;\;\;\;\;\;\;\;\;\;\; \end{array}$$

(14) $$\begin{array}{ccc} {\dot{\Omega }}_{z} & = & {\dot{\Omega }}_{0,z}\sin2\pi \omega_{z}t\;\;\; \end{array}$$

with ${\dot{\Omega }}_{0,x}=10^{-3}$ rad/s${}^{2}$, $\omega_{x}/2\pi$ = 10 mHz, $\phi_{x}$ = 0.5 rad, ${\dot{\Omega }}_{0,z}=3\times 10^{-3}$ rad/s${}^{2}$, and $\omega_{z}/2\pi$ = 20 mHz. Figure 10 summarizes the main results. The top left plot shows the recovered angular accelerations in the time domain over 150 s. The recovered signals agree well with the injected ones. The top right plot shows the linear acceleration in the time domain, which should be ideally zero since linear accelerations were not applied. As in the previous section, the linear acceleration error grows quickly as time increases due to the intrinsic accelerometers’ noise. In addition, oscillatory signals are clearly visible in the three axes of linear acceleration. The bottom plot shows the square-rooted PSD of the linear acceleration, where peaks at 10 mHz and 20 mHz are apparent. In the *x*-axis (blue), only the 20 mHz is visible. In the *y*-axis (red), the 10 mHz and 20 mHz signals are present, and in the *z*-axis (black), only the 10 mHz is visible.

The periodic signals in linear acceleration originate from the coupling between angular velocity and accelerometer noise. For instance, linear acceleration in the *x*-axis is estimated as—cf. Equation (5),

(15) $${\hat{\ddot{r}}}_{x}=\frac{1}{2\sqrt{2}}\sum A_{1,2,5,6}+\ell {\hat{\Omega }}_{y}{\hat{\Omega }}_{z},$$

with

(16) $${\hat{\Omega }}_{y(z)}=\Omega_{y,z}+n_{\Omega_{y(z)}}$$

where $\Omega_{y(z)}$ and $n_{\Omega_{y(z)}}$ are the actual angular velocities around *y* (and *z*) and the noise associated with the angular velocity estimation, respectively. Consequently, the centrifugal term in Equation (15) is

(17) $${\hat{\Omega }}_{y}{\hat{\Omega }}_{z}=\Omega_{y}\Omega_{z}+\Omega_{y}n_{\Omega_{z}}+\Omega_{z}n_{\Omega_{y}}+n_{\Omega_{y}}n_{\Omega_{z}}.$$

The term $n_{\Omega_{y}}n_{\Omega_{z}}$ has been discussed in Section 3.1. $\Omega_{y}\Omega_{z}$ is the *true* correcting term. Thus, the extra noisy terms are $\Omega_{y}n_{\Omega_{z}}$ and $\Omega_{z}n_{\Omega_{y}}$. In this example, the former is zero since $\Omega_{y}=0$, while the latter is (in square-rooted PSD and converted to linear acceleration):

(18) $$\ell S_{\Omega_{z}n_{y}}^{1/2}\left(\omega \right)≃\left\{\begin{array}{cc} \frac{\Omega_{0,z}}{\sqrt{2}\omega }S_{n}^{1/2}\left(\omega -\omega_{z}\right), & \omega =\omega_{z}\\ 0, & \mathrm{otherwise}\; \end{array}\right.$$

where $S_{n}^{1/2}$ is the acceleration noise of a single accelerometer in m s${}^{-2}/\sqrt{\mathrm{Hz}}$. Equation (18) indicates that the accelerometer’s noise at very low frequencies is upconverted to $\omega_{z}=20$ mHz and scaled by the amplitude of the angular velocity $\Omega_{0,z}$. Similarly, the noise in the *y* and *z* axes can be calculated, which leads to the sharps peaks observed in Figure 10 at 10 and 20 mHz for *y*, and 10 mHz for *z*, respectively. For arbitrary angular velocities, the effect is calculated by convoluting the PSDs of the signals and the noise, as shown in Section 3.1.

## 5. Comparison to Other Technologies

**Figure 11:**
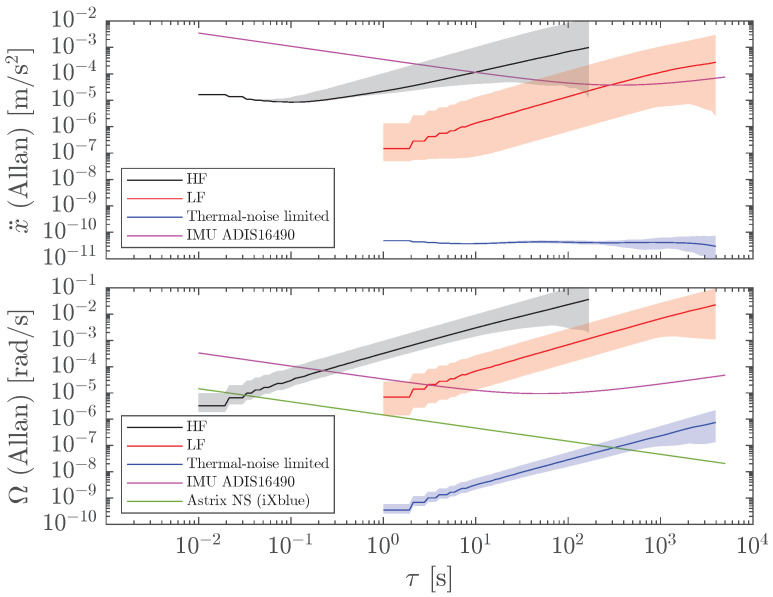
Allan deviation for linear acceleration (**top**) and angular velocity (**bottom**). HF and LF GF-INSs performance are shown in black and red, respectively. A tactical-grade MEMS-based inertial sensor (ADIS16490 from Analog Devices) and an optical gyroscope (Astrix NS from iXblue) are shown for comparison. Blue traces show the performance of an LF GF-INSs limited by the fundamental thermal noise limit of an optomechanical accelerometer.

The benefits of GF-INSs pointed out in Section 1 are useless unless the performance is similar to traditional INSs. In this section, we compare our results to a tactical-grade MEMS-based inertial sensor (ADIS16490 from Analog Devices), and to an optical gyroscope for space applications (Astrix NS from iXblue). These devices report their performance in terms of the Allan deviation; thus, we perform the comparison in such a metric. An obvious advantage of gyroscopes is that they directly measure angular velocity, and, consequently, conversion to orientation requires only one integration step, as opposed to GF-INS, where two integration stages are needed. Figure 11 shows the Allan deviations of the linear accelerations (top) and the angular velocities (bottom) of the HF (black) and LF (red) GF-INSs. The magenta and green traces correspond to the MEMS inertial sensors and optical gyroscopes, respectively. In terms of linear acceleration, the HF GF-INS outperforms the MEMS only from a few 10 ms to a few seconds. The LF GF-INS performance is better than the MEMS up to hundreds of seconds. The situation is different when considering angular velocity. Here, the MEMS inertial sensor and the optical gyroscope are better options than HF and LF GF-INSs. Thus, for the current sensitivity levels of our opto-mechanical accelerometers, GF-INSs have severe limitations, especially for orientation estimation.

However, GF-INS performance is much more promising when we analyze the low-frequency optomechanical accelerometer considering its thermal noise limit. In this case, the expected noise of the accelerometer in Allan deviation is ≃$5\times 10^{-11}$ m s${}^{-2}$ for any integration time [20]. After its propagation through the GF-INS, the linear acceleration and angular velocity precision levels (blue traces in Figure 11) are orders of magnitude better than MEMS-based inertial sensors, and the angular rate of precision is comparable to optical fiber gyroscopes for time scales up to ∼1000 s. Furthermore, the leakage from angular velocity to linear acceleration is non-existent, as shown by the flat blue trace in Figure 11 (top).

Note that the performance levels shown in Figure 11 assume the body is at rest; i.e., only the inherent noise of the accelerometer is taken into account. The excess noise due to actual angular velocity described in Section 4.2 will depend on the dynamics of the application and thus must be estimated for each particular case. However, for thermal-noise-limited accelerometers, this effect is expected to be small.

## 6. Discussion and Summary

In this paper, we have presented the noise analysis for minimal-configuration gyro-free inertial navigation systems based solely on six optomechanical accelerometers. For this analysis, we have only considered the accelerometers’ noise, which has been measured experimentally for our optomechanical accelerometers. The accelerometers are assumed to be perfectly placed in the faces of a cube of size 0.5 m with the sensing direction along the diagonals. This configuration allows calculations for the linear and angular accelerations without having to solve unstable differential equations: the former are a linear combination of accelerometers readings, while the latter require a correcting term derived from the angular acceleration, which introduces noise in the linear acceleration measurement. Consequently, this GF-INS configuration has two drawbacks compared to traditional INSs: (i) angular acceleration is measured instead of angular velocity, and (ii) the non-linear coupling of angular velocity into linear acceleration occurs. The severity of these effects depends on the accelerometer’s noise properties. We have analyzed the performance of gyro-free navigation systems for two types of accelerometers: high-frequency and low-frequency. For the latter, two scenarios have been analyzed: current experimental noise levels and theoretical thermal noise limits.

The results have been compared to commercially available devices: a MEMS-based INS and an optical gyroscope. The outcome indicates that the GF-INS linear acceleration precision levels are on par with the MEMS-based device, while angular information is significantly worse and of little use for navigation. However, when considering the low-frequency optomechanical accelerometer limited by its theoretical thermal noise, $10^{-11}$ m s${}^{-2}$, the results are encouraging: (i) linear acceleration is orders of magnitude better than MEMS and, remarkably, unaffected by angular velocity leakage, and (ii) angular velocity, even after the required extra integration, is significantly better than MEMS and comparable to fiber optical gyroscopes. Finally, the coupling between the actual angular velocity of the body and the accelerometer noise has a mild effect on linear acceleration unless the signal is very large. This effect depends on the application and the expected body dynamics. Nonetheless, a solution to circumvent this issue is by adding another set of six accelerometers with different sensitive directions, which allows linear acceleration to be completely decoupled from angular acceleration.

In summary, we have presented the expected performance of gyro-free inertial navigation units using optomechanical accelerometers. To do so, we have used colored noise models in the frequency domain based on experimental accelerometers’ measurements and propagated them through the gyro-free system. Analytical derivations have been confirmed by simulations. The results show that while the inertial sensors using the current optomechanical accelerometer’s noise levels do not outperform conventional navigation units, the precision when considering the accelerometer’s thermal noise limit can be orders of magnitude better than MEMS systems and comparable to fiber optical gyroscopes.

Future work requires consideration of aspects related to alignment, scaling errors, initial conditions uncertainties, and improvements by adding extra accelerometers or combinations with other sensing technologies. Experimental validation will ultimately determine the performance of gyro-free navigation systems based on linear opto-mechanical accelerometers and the potential benefits for navigation purposes.

## Data Availability

Not applicable.
